# Repeatability of tumor perfusion kinetics from dynamic contrast-enhanced MRI in glioblastoma

**DOI:** 10.1093/noajnl/vdab174

**Published:** 2021-11-22

**Authors:** Ryan T Woodall, Prativa Sahoo, Yujie Cui, Bihong T Chen, Mark S Shiroishi, Cristina Lavini, Paul Frankel, Margarita Gutova, Christine E Brown, Jennifer M Munson, Russell C Rockne

**Affiliations:** 1 Division of Mathematical Oncology, Department of Computational and Quantitative Medicine, Beckman Research Institute, City of Hope, Duarte, California, USA; 2 Division of Biostatistics, Department of Computational and Quantitative Medicine, Beckman Research Institute, City of Hope, Duarte, California, USA; 3 Department of Diagnostic Radiology, City of Hope, Duarte, California, USA; 4 Department of Radiology, Keck School of Medicine, University of Southern California, Los Angeles, California, USA; 5 Department of Radiology and Nuclear Medicine, Amsterdam University Medical Centers, Amsterdam, the Netherlands; 6 Department of Stem Cell Biology and Regenerative Medicine, Beckman Research Institute, City of Hope, Duarte, California, USA; 7 Department of Hematology & Hematopoietic Cell Transplantation, Beckman Research Institute, City of Hope, Duarte, California, USA; 8 Department of Immuno-Oncology, Beckman Research Institute, City of Hope, Duarte, California, USA; 9 Department of Biomedical Engineering & Mechanics, Fralin Biomedical Research Institute, Virginia Tech, Roanoke, Virginia, USA

**Keywords:** brain, DCE, glioblastoma, *K* ^*trans*^, MRI, perfusion, repeatability, QIBA, Tofts model

## Abstract

**Background:**

Dynamic contrast-enhanced MRI (DCE-MRI) parameters have been shown to be biomarkers for treatment response in glioblastoma (GBM). However, variations in analysis and measurement methodology complicate determination of biological changes measured via DCE. The aim of this study is to quantify DCE-MRI variations attributable to analysis methodology and image quality in GBM patients.

**Methods:**

The Extended Tofts model (eTM) and Leaky Tracer Kinetic Model (LTKM), with manually and automatically segmented vascular input functions (VIFs), were used to calculate perfusion kinetic parameters from 29 GBM patients with double-baseline DCE-MRI data. DCE-MRI images were acquired 2–5 days apart with no change in treatment. Repeatability of kinetic parameters was quantified with Bland–Altman and percent repeatability coefficient (%RC) analysis.

**Results:**

The perfusion parameter with the least RC was the plasma volume fraction (*v*_*p*_), with a %RC of 53%. The extra-cellular extra-vascular volume fraction (*v*_*e*_) %RC was 82% and 81%, for extended Tofts-Kety Model (eTM) and LTKM respectively. The %RC of the volume transfer rate constant (*K*^*trans*^) was 72% for the eTM, and 82% for the LTKM, respectively. Using an automatic VIF resulted in smaller %RCs for all model parameters, as compared to manual VIF.

**Conclusions:**

As much as 72% change in *K*^*trans*^ (eTM, autoVIF) can be attributable to non-biological changes in the 2–5 days between double-baseline imaging. Poor *K*^*trans*^ repeatability may result from inferior temporal resolution and short image acquisition time. This variation suggests DCE-MRI repeatability studies should be performed institutionally, using an automatic VIF method and following quantitative imaging biomarkers alliance guidelines.

**Key Points**

- Use of an automatically segmented vascular input function results in more repeatable DCE-MRI analysis in GBM.
- The plasma volume fraction (*v*_*p*_) is the most repeatable perfusion kinetic parameter.
- DCE-MRI studies should follow imaging protocol guidelines set forth by standards bodies such as the Quantitative Imaging Biomarkers Alliance (QIBA) to improve DCE repeatability.

**Importance of the Study**

The Quantitative Imaging Network Glioblastoma Treatment Response (QIN-GBM-TR) dataset includes pretreatment DCE-MRI data with double-baseline MRI acquisitions. As such, this study serves as a benchmark for quantifying the repeatability of tumor perfusion kinetics from DCE-MRI, allowing investigators to set a detection threshold for inferring changes in perfusion attributable to biological changes, rather than technical variations. A key result of this study is the improved repeatability of pharmacokinetic parameters when using an automatically determined VIF in comparison to a manually segmented VIF. We hypothesize that further improvement to reproducibility can be achieved through adherence to QIBA guidelines, notably high temporal resolution and large number of dynamic phases. This has the impact of improving and potentially standardizing DCE protocols for glioblastoma in the future and highlights the importance of institution-specific repeat baseline studies to quantify and improve the reproducibility of DCE analysis.

Dynamic contrast-enhanced MRI (DCE-MRI) is useful for the assessment of patients with brain tumors and enables quantification of perfusion using pharmacokinetic (PK) parameters including the plasma volume fraction ($v_{p}$), extra-cellular extra-vascular interstitial volume fraction$\;(v_{e})$, permeability or exchange rate ($K^{trans}$), and leakage rate ($\lambda^{tr}$).^1,2^ DCE-MRI-derived kinetic parameters (particularly $K^{trans}$ and the ratio $K^{trans}/v_{e}=K^{ep}$) have been shown to be useful imaging biomarkers for glioma grading,^3,4^ predictive of overall survival,^5,6^ and provide early indication of treatment efficacy in high-grade glioma.^7,8^ Despite clear potential for clinical applications, comparisons across institutions and interpretation of changes in DCE-based kinetics are complicated by variations in analysis methods, MRI acquisition, and biological changes in the tissue.

A critical feature of a reliable imaging biomarker is repeatability and reproducibility, as emphasized by guidelines published by the Radiological Society of North America’s Quantitative Imaging Biomarkers Alliance (QIBA).^9^ Observed changes in perfusion kinetic parameters may be due to both measurement error and physiological variations in the tissue. To quantify the repeatability and reproducibility of DCE-MRI based perfusion parameters, it is important to quantify variations not directly associated with changes in biology. Especially in routine clinical practice, or within the context of a clinical trial, it is critical to know whether quantitative differences in a given imaging biomarker represent true biological changes. Quantification of the variability of said biomarker is central to this. Knowledge of this variability can directly impact clinical decision-making where a true biological change may result in changes in patient management. To study the effects of image quality variation alone, patients must undergo repeated, identical MRI studies in a short period of time to minimize the effects of biological changes. From these repeated measurements, a threshold of minimum detectable change in a measurement, typically in terms of percent change, can be derived with statistical confidence.^10^ This threshold of detectable change is known as the percent Repeatability Coefficient (%RC). The smaller the %RC, the more repeatable the measurement, as a change is only statistically distinguishable from measurement error when the change exceeds the %RC^11^.

Several prior studies have evaluated repeatability and reproducibility of DCE-MRI based perfusion parameters in a variety of disease settings.^12–17^ However, to our knowledge, this analysis has not been performed in glioblastoma (GBM) for either the extended Tofts-Kety Model (eTM) model or the Leaky Tracer Kinetic Model (LTKM). Some studies include the Tofts model, analyzing repeatability of *K*^*trans*^, *v*_*e*_,^13^ while some studies include the eTM and analyze only $v_{p}$ in healthy brain tissue.^12^ The Quantitative Imaging Network Glioblastoma Treatment Response (QIN-GBM-TR) dataset with double-baseline DCE-MRI acquisitions is a rare imaging study which allows evaluation of repeatability of perfusion kinetic parameters in GBM.^18^ Investigators have evaluated the percent RCs of dynamic susceptibility contrast (DSC)-MRI PK parameters such as cerebral blood flow (CBF) and volume (CBV) in tumor on this same dataset, and determined that they were 44% and 46% respectively.^19,20^ They also determined that repeatability was increased by normalizing the tumor CBV and CBF values with those of normal tissue. As DCE-MRI typically requires extravasation of contrast agent out of the vessels and into the tissue,^19^ similar normalization methods are not available for brain diseases. Flow measurements such as relative (r)CBV from quantitative DSC imaging do not require a vascular input function (VIF),^21^ and therefore may be more reproducible. However, DSC relies on the assumption that contrast agent does not extravasate from the vessels into the tissue, while DCE requires contrast extravasation for enhancement, and therefore these two modalities provide different, yet complementary information about the perfusion and permeability of the region.^22^

In the present work, we evaluate the repeatability of DCE perfusion kinetic parameters in 29 patients from the QIN-GBM-TR dataset. PK parameters were calculated from two baseline MRI exams taken within 2–5 days of each other, with no change in treatment (Figure 1A). PK parameters were calculated using the eTM model and the LTKM (Figure 1B), with VIFs derived from both automated and manual segmentation methods. By assessing repeatability of DCE-MRI, we aimed to quantify the variation introduced by scanner and computational methods and to inform clinicians and researchers in levels of change detection that are achievable with DCE-MRI, providing an evidence-based threshold for detecting biological changes which may be used to determine disease progression or response to therapy.

**Figure 1.**
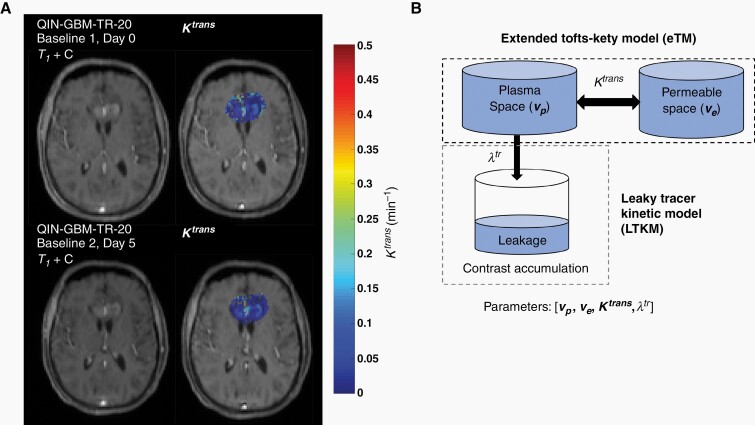
(A) Baseline 1 and 2 (day 0 and day 5) T1-weighted post-contrast DCE-MRI (left) and parametric maps of the perfusion rate constant $K^{trans}$ (right) for patient QIN-GBM-TR-20. (B) The extended Tofts-Kety model (eTM) and the Leaky Tracy Kinetic Model (LTKM) were used to quantify perfusion rate constants *v*_*p*_, *v*_*e*_, *K*^*trans*^, *λ*^*tr*^. The LTKM model includes an additional compartment to the eTM model to account for contrast accumulation which fills at rate $\lambda^{tr}$.

## Materials and Methods

### MRI Data

The data in this publication was sourced from a publicly available dataset, and is used in the present study in accordance to the QIN^18^ and The Cancer Imaging Archive (TCIA)^23^ guidelines. DCE-MRI data were collected from the QIN-GBM-TR study stored in the TCIA public repository between August 2019 and July 2021.^18,24^ The study of publicly available data was approved by the local Institutional Review Board under Exception 4. In the QIN-GBM-TR study, patients underwent two repeated brain MRI scans 2–5 days apart, referred to as baseline 1 and 2, occurring after surgery but prior to the start of therapy. Images were acquired on a single 32-channel Siemens Trio 3T scanners using Siemens 32-channel head coil. The DCE imaging sequence was performed as follows, per study documentation^18^: *T*_10_ mapping was performed by using a 3D FLASH sequence before the injection of contrast agent, with the ratio of repetition time (TR, ms) to echo time (TE, ms) TR/TE = 7.3 ms/4.41 ms, matrix size 128 × 128, field of view (FOV) 230 mm × 230 mm, 20 slices, and 2.1 mm slice thickness. Variable flip angle (VFA) T1 mapping was performed at four different flip angles, 2°, 5°, 10°, and 15°. DCE-MRI utilized a 3D FLASH dual gradient echo sequence with TR/TE1/TE2/α = 6.8 ms/2.61 ms/3.89 ms/10°, matrix size 128 × 128, FOV 230 mm × 230 mm, 20 slices, and 2.1 mm slice thickness. The acquisition was repeated for 60 frames for a total scan time of 6 min, corresponding to a temporal resolution of 6 s. A bolus of 0.1 mmol/kg of Magnevist (Bayer) was injected 52 s after the start of the DCE scan, at a rate of 5 ml/s.^19^

A total of 29 double-baseline DCE-MRI image pairs were included in this study. From the 54 patients in the QIN-GBM-TR study, nine patients did not have complete VFA images to compute *T*_10_ mapping, three patients were not found on the TCIA archive, four patients lacked one of the baseline imaging time points, two patients images contained motion artifacts, four patients lacked significant contrast enhancement, two patients flip angle images did not cover the tumor region, and one patient had a tumor at the base of the skull and had no sagittal sinus in the field of view to use for a VIF (Supplementary Table 1).

### Identification and Calculation of the Vascular Input Function

**Figure 2.**
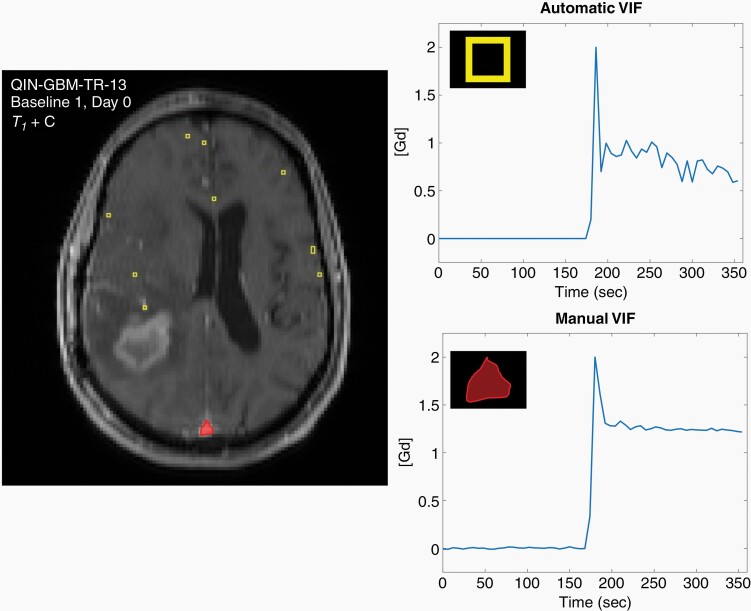
Two methods to identify and calculate the VIF were used in this study. Baseline 1 for study number QIN-GBM-TR-13 was chosen to highlight differences between the two VIF segmentation methods. The first method determines the VIF automatically with an algorithm which selects only voxels (yellow boxes) with a rapid change in signal intensity and short time-to-peak. The second, more common method, is manual segmentation of the superior sagittal sinus (red region). Both methods average the signal intensity of all voxels to create a composite profile. The automatic VIF captures contrast washout and the initial peak, in contrast to the manual VIF in the sagittal sinus, which in this case shows a rapid saturation of signal with smaller initial peak and slow washout.

The VIF was identified and calculated with two different methods: automatic and manual segmentation (Figure 2). The automatic segmentation algorithm selects voxels with rapid signal enhancement, defined as the difference between bolus arrival and peak time less than 10 s, and eliminates voxels with a maximum signal intensity below the 90th percentile in the whole image.^25^ The manual segmentation method used a user-defined region of interest (ROI) including the superior sagittal sinus, drawn on 3–5 of the most central slices of the field of view.^8^ Both the automatic and manual VIF methods averaged the signal intensity and measured pre-contrast absolute *T*_1_ (*T*_10_) of all selected voxels to create a composite profile and were computed for each DCE scan.^25^ To directly compare perfusion parameters calculated from the automatic and manual approaches, the VIF was normalized to the maximum of the automatic and manual VIFs, such that the maximum value of the VIF for each patient is equal to 2. This methodology was selected to directly compare relative VIF concentration as opposed to absolute VIF concentration.^25^

### Perfusion Kinetic Parameter Estimation

A map of $T_{10}$ was estimated using the VFA method.^26^ All flip angle images were registered to the first DCE-MRI image before *T*_10_ quantification. The estimated *T*_10_ map was used to generate the concentration time profile.^27^ The VIF signal-intensity profile was corrected for $T_{2}^{*}$ dephasing using dual-echo DCE sequence and the appropriate dual-echo correction.^28^ Voxel-wise concentration was calculated from *T*_10_ and signal-intensity as:


$$\begin{array}{l} \frac{1}{r_{1}}\left(\frac{1}{T_{1}\left(t\right)}-\frac{1}{T_{10}}\right)=C(t), \end{array}$$ (1)

where


$$\begin{array}{l} \frac{1}{T_{1}\left(t\right)}=-\frac{1}{TR}\ln\left(\frac{1-A}{1-A\;cos\alpha }\right), \end{array}$$ (2)

$$\begin{array}{l} A=\frac{S\left(t\right)}{S\left(0\right)}\frac{1-exp(-TR/T_{10})}{1-\exp\left(-TR/T_{10}\right)cos\alpha }, \end{array}$$ (3)

*r*_1_ is the relaxivity of the contrast agent, *T*_1_(*t*) is the *T*_1_ of the signal intensity at time *t*, α is the flip angle, and TR is the repetition time.^29^ Two PK models were used for the estimation of perfusion parameters: the eTM^1^ (Eq. 4) and the LTKM (Eq. 5).^2^ The mathematical formulation of the models are as follows:


$$\begin{array}{ll} & C\left(t\right)=v_{p}C_{p}(t)+K^{trans}\underset{0}{\overset{t}{\int }}\;C_{p}\left(\tau \right)\;\;\;exp\left(-\frac{K^{trans}}{v_{e}}\left(t-\tau \right)\right)d\tau \end{array}$$ (4)

$$\begin{array}{ll} & C\left(t\right)=v_{p}C_{p}(t)+K^{trans}\underset{0}{\overset{t}{\int }}\;C_{p}\left(\tau \right)\;\;\;exp\left(-\frac{K^{trans}}{v_{e}}\left(t-\tau \right)\right)d\tau +\lambda^{tr}\underset{0}{\overset{t}{\int }}\;C_{p}\left(\tau \right)d\tau \end{array}$$ (5)

where C(t)is the concentration of contrast agent for an individual voxel at time t, $v_{p}$ is the volume fraction in the plasma, $C_{p}(t)$ is the concentration in the plasma given by the VIF, $K^{trans}$ is the perfusion rate constant, $v_{e}$ is the volume fraction in the extra-cellular, extra-vascular space, and $\lambda^{tr}$ is the leakage rate constant (LTKM only). Model parameters were estimated with the Levenburg–Marquardt regression method. The procedure for calculating eTM model parameters from a given concentration curve $C_{p}(t)$ was validated with the QIBA DCE-MRI digital reference object (DRO)^30^ (Supplementary Figures S3 and S4).

## Goodness of Fit Criteria

Goodness of model-data fit was quantified with the $R^{2}$ statistic. Given data values $C_{i}$, *i* = 1, 2,..., *N*, with mean $\bar{C}$ and model values $f_{i}$, goodness of fit is given by $R^{2}=1-\frac{SS_{err}}{SS_{tot}}$, where $SS_{err}=\underset{i}{\sum }\;{\left(C_{i}-f_{i}\right)}^{2}$ is the residual sum of squares, and $SS_{tot}=\underset{i}{\sum }\;{\left(C_{i}-\bar{C}\right)}^{2}$ is the total sum of squares. The value of $R^{2}$ lies between 0 and 1, where a value of 1 indicates perfect model-to-data agreement. Voxels with $R^{2}\leq 0.5$ were considered to have a poor fit and are subsequently excluded from analysis. The number of voxels with fitting confidence level $R^{2}>0.5\;$ were used as an estimation of the tumor volume. Voxels below this threshold are typically necrotic voxels with low contrast enhancement and low signal-to-noise ratio. Voxels which did not fit the model to a confidence level *R*^*2*^ > 0.5 were excluded from statistical analysis, as contrast enhancement is required for accurate model parameterization.^25^

### Statistical Analysis

The tumor region was manually segmented over all slices in the 3D volume to include the contrast-enhancing lesion and surrounding tissue. Mean parameter values of the voxels within the ROI with fitting confidence $R^{2}>0.5\;$ were included in statistical analysis. The relative change between baseline scans for each patient was calculated as $d=\ln\left(\frac{P_{B2}}{P_{B1}}\right)$, where $P_{B1}$, $P_{B2}$ are parameter values for baseline 1, and 2 images, respectively. Repeatability was assessed with Bland–Altman analysis.^10,27^ The Bland–Altman 95% limits of agreement (LoA) is LoA = *d* ± 1.96σ where σ is the standard deviation (SD) of the distribution.^10,11,31^ The RC at 95% confidence is given by $RC=1.96\;\sqrt{2}\;wCV$, where, $wCV=\sqrt{\frac{1}{n}\underset{j=1}{\overset{n}{\sum }}\;\frac{1}{2}{\frac{\left(x_{j1}-x_{j2}\right)}{{\bar{x_{j}}}^{2}}}^{2}}\;$, $x_{j1}$ and $x_{j2}$ are the parameter values at baseline 1 and baseline 2 respectively, and $\bar{x_{j}}$ is the mean parameter baseline value for patient *j*. The coefficient of variation (CoV) for the mean difference of each parameter is calculated as $CoV=\sqrt{\frac{1}{n}\underset{j=1}{\overset{n}{\sum }}\;\frac{s_{j}}{\bar{x_{j}}}}$ where $s_{j}$is the standard deviation of the two measurements for patient *j*.^13^ Percent RC is given by %RC = RC×100 as in Peled et al.^11^ The change of whole brain *T*_10_ map histograms between baseline scans is calculated as *T*_10_ Shift $=\;\frac{P1-P2}{P1}$, where *P*_*1*_ is the histogram peak of the *T*_10_ values at baseline 1, and *P*_*2*_ is the histogram peak of the *T*_*10*_ values at baseline 2.

## Results

**Figure 3.**
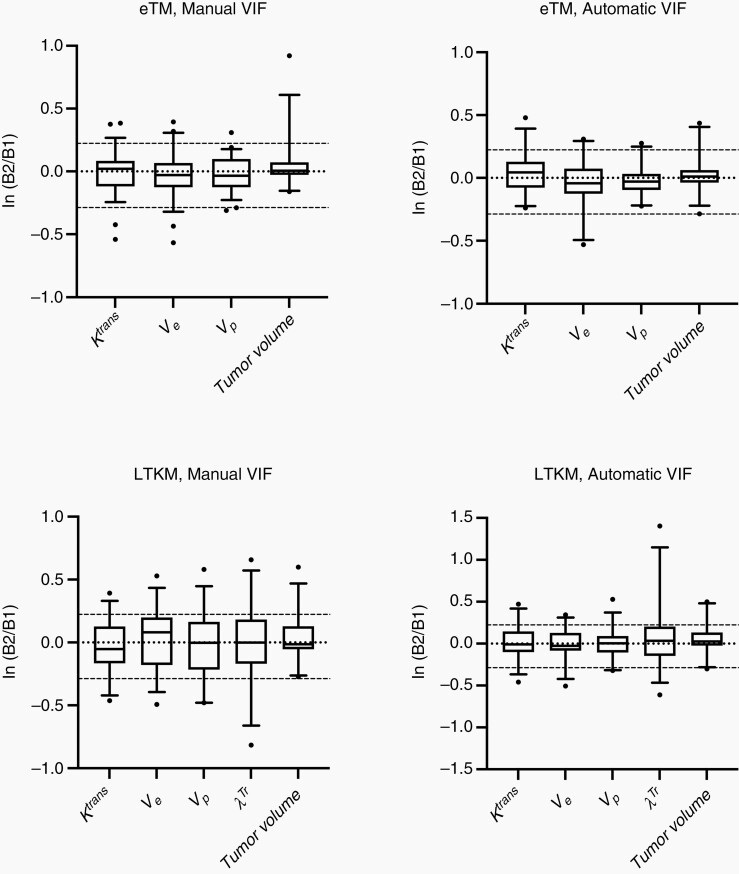
Distributions of relative change between baseline 1 and 2 scans for natural log transformed perfusion parameters and tumor volume for eTM and LTKM models with manual and automatically determined VIF methods for all 29 double-baseline image sets. Box bounds show inner quartiles (25%–75% percentiles), and whiskers extend to the outer quantiles (5%–95% percentiles). Dashed lines are placed at zero, indicating exact repeatability, and ln(1.25) and ln(0.75), denoting the QIBA guidelines for standards of ±25% limits of agreement.

The relative change of kinetic parameters for both the eTM and LTKM for all 29 patients/image pairs is shown in Figure 3. For both models and VIF methods, the inner quartiles (25–75th percentile) for all parameters are less than ±25%, as suggested by QIBA guidelines.^9^ There was no significant difference found between tumor size for either eTM or LTKM, indicating both models fit equally well to the data (Table 1 and Figure 3). Smaller variation from baseline 1 to 2 is observed in the kinetic parameters derived with the automatic VIF, as compared to manual VIF for both eTM and LTKM models. Bland–Altman plots for each parameter derived from eTM and LTKM with automatic VIF are given in Supplementary Figures S1 and S2. Similar analysis was performed by a second observer, with DRO validation and inter-observer comparison provided in the supplementary materials (Supplementary Figures S5, S6 and Supplementary Table S7). The LoA and bias from Bland–Altman analysis, %RC, and CoV are given in Table 1. For the interested reader, mean parameter values for all model and VIF combinations are included in the supplementary materials (Supplementary Table S11).

**Table 1.** Summary of Bland–Altman Repeatability Analysis of Perfusion Parameters for eTM and LTKM Models With Automatic and Manual VIF Methods. Limits of agreement are expressed as the lower bound (LB) and upper bound (UB)

Parameter	Bias ln(*B*_2_) −ln(*B*_1_)	1.96*σ* LoA (LB, UB)	%RC (95%)	%CoV
**eTM, automatic VIF**				
Size (*n*-vox)	8.2E-2	−0.58, 0.74	64	33
*v*_*p*_	−4.9E-2	−0.59, 0.49	53	32
*v*_*e*_	−7.9E-2	−0.95, 0.79	82	40
*K* ^*trans*^ (min^−1^)	8.1E-2	−0.67, 0.83	72	39
**eTM, manual VIF**				
Size (*n*-vox)	0.14	−0.77, 1.0	76	33
*v*_*p*_	−5.0E-2	−0.73, 0.63	66	37
*v*_*e*_	−7.7E-2	−1.0, 0.87	88	42
*K* ^*trans*^ (min^−1^)	−6.4E-3	−0.90, 0.89	83	39
**LTKM, automatic VIF**				
Size (*n*-vox)	0.11	−0.69, 0.91	76	36
*v*_*p*_	−5.6E-3	−0.77, 0.77	72	37
*v*_*e*_	−3.7E-4	−0.87, 0.87	81	41
*K* ^*trans*^ (min^−1^)	−3.8E-2	−0.84, 0.91	82	41
*λ* ^*tr*^ (min^−1^)	0.18	−1.5, 1.9	1.3E2	50
**LTKM, manual VIF**				
Size (*n*-vox)	0.08	−0.73, 0.89	76	38
*v*_*p*_	−6.2E-2	−1.2, 1.1	1.1E2	47
*v*_*e*_	6.6E-2	−1.0, 1.1	1.0E2	48
*K* ^*trans*^ (min^−1^)	−8.0E−2	−1.0, 0.86	89	43
*λ* ^*tr*^ (min^−1^)	−8.0E-3	−1.4, 1.4	1.3	51

**Figure 4.**
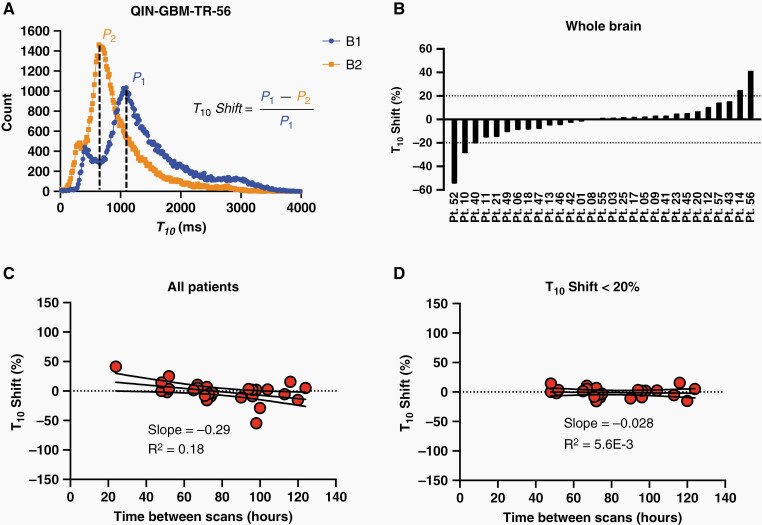
(A) Histogram of *T*_10_ for the whole brain for patient QIN-GBM-RT-56 indicating a *T*_10_ shift between baseline 1 and 2 scans. (B) *T*_10_ shift for all 29 patients. Pt. X represents MRI data set identifier QIN-GBM-TR-X. (C) Correlation between *T*_10_ shift and time between baseline scans. (D) For patients with *T*_10_ shift < 20%.

The %RC was lowest in $v_{p}$ for both the LTKM (72%) and eTM (%RC = 53%) with automatic VIF. The range of absolute *T*_10_ shift (whole brain) between baseline 1 and 2 scans was (11±12)% (mean±SD) with maximum of 55%. Out of 29 patients, four patients, *T*_10_ shift > 20% were observed, corresponding to a significant *B*_1_ field inhomogeneity effect (Figure 4). There was no significant correlation found between *T*_10_ shift and time between baseline scans. Parameter values for patients with *T*_10_ shift > 20% fall within the Bland–Altman LoA, except for one patient which is an outlier in *K*^*trans*^ (eTM, auto VIF, Table 1, Supplementary Figure S1), and leakage rate $\lambda^{tr}$ (LTKM, manual VIF, Table 1, Supplementary Figure S2). The CoV was lowest for all parameters under eTM, and automatic VIF determination, with a standard deviation of the relative change of 41%, 41%, 37% for *K*^*trans*^, *v*_*e*_, and $v_{p}$ respectively (Supplementary Tables S1 and S7). For nearly all cases, the LoA are smaller for the automatic VIF, as opposed to manual VIF (Table 1). All VIF methods and model parameterizations are ranked by CoV in Supplementary Table S8.

## Discussion

As a growing number of clinical trials are collecting perfusion rate constants and other biomarkers from DCE-MRI, it is imperative that the repeatability of these parameters is assessed in vivo to establish the threshold above which a true biological change may be detected due to tumor progression or effect of therapy. In GBM, the perfusion rate constant $K^{trans}$ has been shown to be indicative of blood-brain barrier (BBB) breakdown, and is thought to be an indicator of therapeutic response.^8^ The results presented in this work demonstrate repeatability of perfusion parameters over a short timeframe providing an estimate of change required to attribute changes to biological or physiological changes in the patient.

Similar analysis was performed by Jafari-Khouzani et al.^19^ and Prah et al.^20^ on the DSC portion of the same dataset.^18^ The authors reported a %RC of 46% for CBV, and 44% for CBF in enhancing tumor after normalization to healthy tissue using a gradient echo sequence, while healthy white matter was found to be more repeatable with an %RC of 8% for both CBV and CBF.^19^ In contrast, for DCE-MRI the lowest %RC were found to be 72% for the LTKM and 53% for the eTM for $v_{p}$ using an automatic VIF. The %RC of *K*^*trans*^, the most comparable measurement between DCE- and DSC-MRI, as *K*^*trans*^ is a mixed measure of permeability and perfusion,^1^ for eTM using automatic VIF was determined to be 72%. It is important to note that no comparison or normalization between tumor and healthy tissue can be made for DCE-MRI in the brain, as the method utilized in the QIN-GBM-TR dataset requires contrast extravasation for *T*_1_ signal enhancement, which is difficult to observe for healthy brain tissue with intact BBB.^32^

A key finding of this study is the improved consistency of perfusion parameters between double-baseline scans when using an algorithm to automatically identify and calculate the VIF, as opposed to a VIF obtained by manual segmentation of the superior sagittal sinus. As the sequence used for this analysis is dual-echo, the calculated contrast concentration is more susceptible to *T*_2_* effects.^28^ Corrections performed to cancel these effects may not completely account for flow induced *T*_2_* effects. *T*_1_ signal intensity may also be artificially enhanced in large vessels due to arrival of spin-labeled blood from outside of the imaging plane.^33^ The automatic VIF method tends to identify small voxels associated with smaller vessels and capillaries, rather than large arteries or veins which are more susceptible to *T*_2_* and flow artifacts and may be a better representation of the perfusion local to the tumor tissue. In the case of the brain, and in this study in particular, the only sufficiently large vessel present in the field of view for all patients was the superior sagittal sinus, which is a vein and therefore not the most suited vessel for an input function, as the dural sinus may become saturated without a definite peak value. Large vessels may also be susceptible to partial volume effect if ROIs are drawn too close to the vessel boundary.

In our analysis, the %RC of *K*^*trans*^ and $v_{p}$ for both the eTM and LTKM when using an automatic VIF is less than the %RC calculated from a manual VIF segmentation. Previous studies have shown that the quality of DCE time-course fitting is highly dependent on VIF selection.^11^ Much work has been done to develop smooth, parameterized population VIFs, which offer the benefit of improved repeatability at the cost of decreased measurement accuracy for the individual.^34^ Therefore, we recommend the usage of an automatic approach, similar to that used in the present study,^25^ for maximal consistency and minimizing the risk of accuracy loss due to the use of a population VIF.^34^ Additionally, we also recommend converting MR signal intensity curves to contrast agent concentration curves and fitting to these, as accurate PK model fitting to DCE-MRI signal intensity data requires careful correction for internal consistency.^35^

In an effort to increase the fidelity of measurements made by DCE-MRI, the QIBA makes methodology recommendations for maximizing the quality of DCE-MRI acquisitions. Among these recommendations include the use of a 3T magnet, a field of view between 220 and 240 mm with an acquisition grid of 256 × 128–160, a 3D spoiled gradient recalled MRI sequence, with at least five pre-contrast and 40 post-contrast phases acquired with < 10 s temporal resolution.^29^ From these recommendations, the QIBA advises a repeatability of 21% in *K*^*trans*^ parameters as a threshold for physiological change detection.^29^ In contrast, the present QIN-GBM-TR dataset was acquired using a 3D FLASH dual gradient echo sequence, with a field of view equal to 230 mm × 230 mm on a 128 × 128 grid, with roughly 30 pre-contrast phases and 30 post-contrast phases,^18^ or roughly 3 min of contrast dynamics. According to the QIBA DCE-MRI guidelines, this dataset is both spatially and temporally under-sampled in post-contrast and is acquired with a sequence requiring *T*_2_* decay correction. Temporal under-sampling known to poorly affect the accuracy of *K*^*trans*^ estimation,^8^ and fit accuracy is decreased with few dynamic acquisitions.^36^ As *K*^*trans*^ measurement is highly sensitive to sampling rate and the ability to capture the peak of the VIF,^36^ we recommend that studies aimed at measuring *K*^*trans*^ accurately and repeatably, do so in accordance with the QIBA recommendations.

The present work demonstrates the %RC of *K*^*trans*^ and $v_{e}$ to be 72% and 87%, respectively, using the eTM model with automatic VIF determination in the QIN-GBM-TR dataset. A prior study of DCE-MRI parameters found that the %RC for *K*^*trans*^ and $v_{e}$were 7.7% and 6.2% in patients with newly diagnosed glioma.^13^ We hypothesize that a dataset acquired under full accordance to the QIBA guidelines would have smaller CoVs and RCs, similar to those presented by Jackson et al.^13^ Because GBM is a rapidly progressing disease,^37^ it is possible that tumor progression may contribute to changes in perfusion parameters in the 2–5 days between double-baseline scans.^18^

The repeatability of DCE-MRI may be affected by both image acquisition and post-processing. Image acquisition includes signal-to-noise ratio, flip angle accuracy, *B*_1_-inhomogeneity, temporal and spatial resolution, length of imaging, and motion artifact.^38^ Post-processing includes the applicability of the applied kinetic model, accuracy in VIF detection and deconvolution, accuracy of selected anatomic ROIs to the perfusion maps. In particular, *T*_10_ mapping from VFA methods has been shown to have a large inter-site variability (30%–40%) when using a phantom.^15^ These variations are thought to be due to *B*_1_ field inhomogeneity, and contribute strongly to differences in the quantification of contrast agent concentration.^15^ MR-Fingerprinting may aid in increasing the repeatability of pre-dynamic imaging, and has been shown to be highly repeatable, with RCs < 2% for *T*_10_ mapping and less than 10% for *T*_2_ mapping.^39^ In our analysis, four subjects with a large mean %*T*_10_ shift were identified but did not demonstrate large changes in perfusion parameters. However, these patients were identified as outliers for tumor volume calculations, suggesting worse model-to-data fitting in the tumor region. As baseline *T*_10_ maps are acquired, the authors believe that *T*_10_ variations, potentially due to contrast accumulation are accounted for, but should be carefully studied on an individual basis, and compared to the remaining population to rule out inconsistencies. As GBM are highly heterogeneous, fitting to nested versions of the eTM or LTKM may lead to increased fit accuracy, and granular model identification may contribute to increased parameter repeatability.^40^

In a multi-institutional study of DCE-MRI perfusion parameter calculation, it was shown that parameter quantification is highly dependent on the model-data fitting algorithm.^41^ There was wide agreement between parameters on a DRO with no noise present, but upon the addition of noise, the disagreement between institutions became apparent. In general, the authors found that trends in parameter changes (ie, increasing *K*^*trans*^) were consistent between sites, but the raw parameter values saw errors up to 100% of the true DRO parameter value in the presence of noise. In a similar study designed to assess the inter-reader variability of DCE-MRI parameters in GBM, the inter-reader variation in *K*^*trans*^ values was determined to be 27% ± 34% (mean ± SD), and within-patient CoV reaching up to 65% in post-treatment cases.^42^ We therefore strongly support further development and validation of standardized DCE-MRI quantification software package by standards bodies, such as the National Institute for Standards in Technology or QIBA, and further collaboration in reporting best-practices, to maximize inter-site communication and improve repeatability in DCE-MRI protocols and analysis. Until such a standard is developed, we recommend using the same reader and quantification algorithms to remain consistent within studies performed at an individual site. Due to variations in compartmental PK models used to process DCE-MRI data for varying MR acquisitions, organs, diseases, and VIF determinations, or until a standard is accepted and made widely available, the authors strongly recommend that each site acquire double-baseline data and perform similar test-retest analysis whenever possible, to quantify the detection threshold for biological changes in patients.

There were several limitations to this study. First, it was not possible to control for potential confounding variables in imaging acquisition, such as different imaging technologists, infusion protocols, patient biological variability, and protocol design. Second, we did not assess the non-enhancing FLAIR signal hyperintense tumors while focusing on the contrast-enhancing portion of the tumor for DCE analysis. The non-enhancing portion of the tumor is important in assessing treatment response and has been incorporated into the updated criteria for Response Assessment in Neuro-Oncology.^43,44^ DSC-MRI may generate rCBV measurements to assess both enhancing and non-enhancing tumor, and it does not rely on the VIF determination, potentially making the method more robust in this regard. However, much work has been done to show the unique, yet complementary capabilities of DCE and DSC methods to assess tumor microvascular hemodynamics.^45^ Third, our study was limited in lacking follow-up data for the post-chemoradiation phase of the disease. Finally, low *K*^*trans*^ repeatability in our analysis may result from inferior temporal resolution and short duration of the DCE-MRI acquisition as compared to the QIBA recommendations.^9^ Temporal under-sampling and limited dynamic acquisitions are known to poorly affect the accuracy of *K*^*trans*^ estimation.^8,36^ DCE imaging quality is critical for advancing tumor assessment methods in clinical trials designed to use DCE parameters (such as *K*^*trans*^) as end points. The recently published ACRIN 6686 trial showed rCBV, but not *K*^*trans*^, to be a sensitive biomarker of early biological changes following bevacizumab treatment in newly diagnosed GBM.^46^ More work is needed to further characterize tumor vasculature with both perfusion and permeability parameters in response assessment of GBM.

## Conclusion

Using a publicly available dataset with double-baseline DCE-MRI acquired within 2–5 days of initial imaging, the repeatability of DCE-MRI perfusion rate constant parameters was measured in 29 GBM patients, using both automatic and manual methods for VIF determination. For the eTM model, the %RCs for the parameters were determined to be 72%, 82%, and 53% for $K^{trans}$, v, and $v_{p}$, respectively. For the LTKM model, the %RCs were determined to be 72%, 81%, 82%, and 130% for *v*_*p*_, *v*_*e*_, *K*^*trans*^, and $\lambda^{tr}$, respectively. The practical value of our analysis is summarized as follows: first, this study demonstrates that automated VIF determination can increase repeatability in DCE analysis. The automatic approach also simplifies clinical implementation, as it is less time consuming and less demanding on dedicated research personnel as compared to the manual segmentation. Second, this study indicates the need for standardization of the DCE-MRI protocols to facilitate comparison across institutions and multi-center trials. Finally, the establishment of RCs and coefficients of variation in a standardized dataset may allow researchers to establish an evidence-based change detection threshold, which may be updated with the investigators’ own DCE analysis methodology and MRI equipment. Due to suboptimal spatial and temporal sampling in this image set, we posit that the %RCs presented here constitute likely a worst-case, upper bound for the detection threshold of biological and physiological changes in GBM under identical imaging conditions. We hypothesize that repeatability of DCE-MRI quantification will be improved, including yielding smaller %RCs, through adherence to standard imaging guidelines such as those suggested by QIBA.

## Supplementary Material

vdab174_suppl_Supplementary_Matieral

vdab174_suppl_Supplementary_Data

## Notes

**Authorship statement**. Conceptualization: R.C.R. Data Curation: P.S., R.T.W., R.C.R. Formal Analysis: Y.C., P.F., P.S., R.T.W. Funding Acquisition: R.C.R., C.E.B., J.M. Investigation: R.T.W., P.S., R.C.R. Methodology: R.C.R., Y.C., P.F., P.S., R.W. Project Administration: R.C.R. Resources: R.C.R., C.E.B., J.M. Software: R.T.W., P.S. Supervision: R.C.R., P.F., J.M. Validation: R.T.W., P.S., R.C.R., Y.C., C.L., P.F., M.G., M.S.S., B.T.C. Visualization: R.T.W., R.C.R., P.S. Writing—Original Draft Preparation: P.S., R.T.W., R.C.R. Writing—Review and Editing: all authors.
