# Supplementary material for: Repeatability of tumor perfusion kinetics from dynamic contrast-enhanced MRI in glioblastoma
Source: Neurooncol Adv. 2021 Nov 22;3(1):vdab174. doi: 10.1093/noajnl/vdab174 (PMC8715899; doi:10.1093/noajnl/vdab174)
Supplement: vdab174_suppl_Supplementary_Matieral [file vdab174_suppl_supplementary_matieral.docx]

# **Supplementary Material**

## **Factors influencing perfusion parameter repeatability**

Both experimental and post-processing factors influence the repeatability of perfusion parameters from DCE-MRI data. Experimental factors include those introduced by the scanner, patient, and imaging protocol, for example, B1 field inhomogeneity, patient motion in the scanner, tumor size, imaging parameters such as slice thickness, or the patient’s weight. Postprocessing factors include selection of VIF, pharmacokinetic model, image registration, and segmentation of the tumor ROI. The effect of B1 inhomogeneity was explored by calculating the shift between histograms peak of pre-contrast $T_{10}$ maps computed for test and retest images using $T_{10}shift\left( \% \right)=\frac{{P_{1}-P}_{2}}{P_{1}} \times100$. The Pearson correlation coefficient, R^2^ value, and p-value of each test were calculated to determine if there was significant (p < 0.05) and strong correlation (R^2^ > 0.5) between T_10_ shift and parameterization change. No such strong correlations were found in this dataset, but the authors encourage those performing similar research to examine and consider factors such as T_10_ shift in their analysis of DCE-MRI datasets.

## **Inter-reader variability**

DCE analysis using the 2-compartment Tofts-Kety model was performed by two separate observers: Observer 1 (presented in main text) and Observer 2 (included in supplement). The observers quantitatively validated their eTK values using the QIN/Barboriak DCE digital reference object, and qualitatively validated their pipelines using two patients. It was determined through these analysis that each pipeline is capable of fitting the same parameters in a high signal, low noise environment, while the observations made by each observer diverge upon examination of noisy real-world data. To the authors’ knowledge, the primary difference between the methods of Observer 1 and Observer 2 are the enforcement of bounds on compartment volume fractions. Observer 1 allows for the fitting of volume fractions (specifically, *v_e_*) to be greater than unity, and removes such voxels from the analysis. Observer 2 enforces bounds on volume fractions between 10^-6^ and 1 during the curve-fitting routine.

## **Correlation Testing on Bland-Altman Results**

For each Bland-Altman plot, a Pearson correlation test was performed between the mean of baseline scans (x-axis) and difference between baseline scans (y-axis). This analysis was performed on all parameters, and tumor volume, for both VIF methodologies. There was no strong correlation (*R^2^* > 0.50) found for any parameters or conditions. This suggests all model parameters are independent of time between scan (within the 2-5 day range in the dataset) and tumor size. However, significant (*p* < 0.050) correlations were found. For parameters derived from the manual VIF eTM, a significant correlation was found between mean tumor volume and difference in tumor volume (*r* = -0.56, *p* < 0.01). Correlations between difference and mean were also found for *K^trans^* under the automatic VIF eTK model (*r* = -0.43, *p* = 0.02), and automatic VIF LTKM model (*r* = -0.40, *p* = 0.03), as well as for leakage in the automatic VIF LTKM model (*r* = -0.55, *p* < 0.01).***Supplementary Table 1:*** *Patients from QIN-GB-TR treatment response database analyzed in this study.*

| QIN GBM treatment response study patient selection | |
| --- | --- |
| Total Patients in QIN-GBM-RT study | 54 |
| No re-test data | -4 |
| Not available on TCIA | -3 |
| Incomplete flip angle images | -9 |
| Motion artifacts | -2 |
| No visible contrast enhancement | -4 |
| Pre-contrast T1 and DCE slice mismatch | -2 |
| Tumor at base of skull | -1 |
| **Total patients included** | 29 |

**Figure S1.** Bland-Altman repeatability analysis, which plots the average versus difference in natural log transformed parameter values from baseline 1 and 2 scans for the extended Tofts-Kety model for two VIF methods. Dashed lines indicate the 95% limits of agreement. Blue points indicate 4 patients with a T_10_-shift between baseline 1 and 2 with fold-change greater than 0.20.

**Figure S2.** Bland-Altman analysis plotting average versus difference in natural log transformed parameter values from baseline 1 and 2 scans for the leaky tracer kinetic model for two VIF methods. Dashed lines indicate the 95% limits of agreement. Blue points indicate 4 patients with a T_10_-shift between baseline 1 and 2 with fold-change greater than 0.20.

**Figure S3.** QIN/Barboriak DRO validation for 2 observers. Validation of the extended Tofts Kety model was performed using the QIN/Barboriak digital reference object. On the top row, the nominal parameter from the digital reference object (left) is compared to the parametric fit from Observer 1 (right) (data in main text). The same analysis is performed for Observer 2, and the results are shown on the bottom row. For each observer, *v_p_* is fit with the least error, followed by *K^trans^*. *v_e_* is the least accurate parameter for both observers, with inaccuracies occurring at low values of *K^trans^* and *v_e_*.


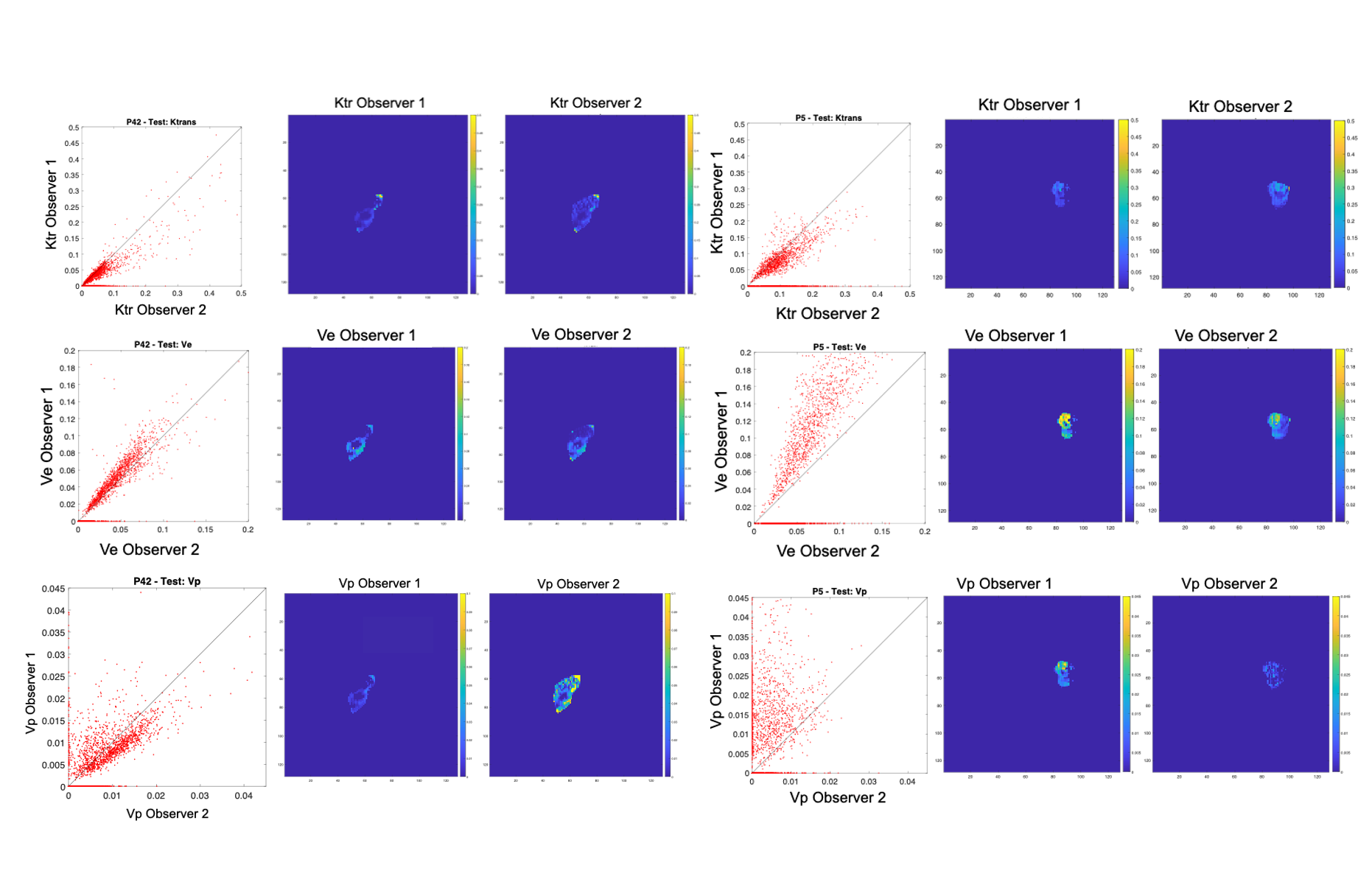


**Figure S4.** Inter-observer validation on individual voxels. In addition to utilizing the DRO, the parametric maps of the extended Tofts Kety model were directly compared between Observers 1 and 2. In each scatter plot, the parameters *K^trans^*, *v_e_*, and *v_p_*, calculated by both observers are plotted and compared to the line of unity. Patient 42 had the best voxel-wise agreement (points lie closest to the line of unity), while Patient 5 had the worst. For patient 5, the observations from Observer 2 tended to fit the parameter *v_p_* closer to 0, possibly because of poor determination of the bolus arrival time due to low SNR. Thus, the authors hypothesize that the parameter *v_e_* is artificially high for Obs. 2 in order to compensate.

**Figure S5.** Bland-Altman plots of extended Tofts-Kety model parameters (Observer 2) after natural log transformation. Dashed lines indicate the 2-standard deviation limits of agreement. Blue points indicate patients with a T_10_-shift fold-change greater than 0.20.

**Figure S6.** Distribution of parameter changes for observer 2 for eTM model. Box-and-whisker plots of the ln(fold-change) (Observer 2) from test to retest of each parameter, for each model and VIF method. Box bounds lie on the 75% quantile, and whiskers extend to the 95% quantile. Outliers are excluded. Dashed lines are placed at ln(1.25) and ln(0.75), denoting the QIBA guidelines for standards of the limits of agreement*.*

**Table S7.** Observer 2 Bland-Altman statistics.

| **Parameter** | **Bias**  **ln(B2)-ln(B1)** | $\boldsymbol{1.96}\boldsymbol{\sigma}$**(95%) LoA**  **(LB, UB)**  **ln(B2)-ln(B1)** | **%RC (95%)** | **CoV%** |
| --- | --- | --- | --- | --- |
| **eTM – auto VIF** | | | | |
| **Size (n-vox)** | 9.8E-2 | -0.60, 0.79 | 60 | 28 |
| ***v_p_*** | 0.10 | -1.8, 2.1 | 1.6E2 | 60 |
| ***v_e_*** | 5.8E-2 | -1.2, 1.3 | 1.2E2 | 49 |
| ***K^trans^* (min ^-1^)** | 0.29 | -1.2, 1.8 | 1.4E2 | 54 |
| **eTM – manual VIF** | | | | |
| **Size (n-vox)** | 9.7E-2 | -0.60, -0.80 | 61 | 28 |
| ***v_p_*** | 0.41 | -2.5, 3.3 | 2.2E2 | 68 |
| ***v_e_*** | 0.13 | -1.1, 1.4 | 1.2E2 | 50 |
| ***K^trans^* (min ^-1^)** | 7.7E-2 | -1.4, 1.5 | 1.3E2 | 51 |

**Table S8.** Ranking of parameters by Coefficient of Variation, model, observer, and VIF method

**Figure S9.** Determination of goodness-of fit criteria via *R^2^* thresholding. If all voxels within the segmented volume are included in the analysis, the histogram of *v_p_* (as well as *K^trans^*) is left-shifted to 0, including healthy and necrotic tissue, whereas the measurement of perfusion parameters is desired in perfused tumor tissue, with degraded BBB. Above are shown histograms of the parameter *v_p_*, given two thresholds of *R^2^* cutoff (*R^2^* > 0.5 & *R^2^* > 0.8). A threshold of *R^2^* > 0.5 was selected as a balance between rejecting voxels containing necrotic/healthy, and biasing analysis towards those voxels with highest perfusion.

**Table S10:** File paths for Baseline 1 and Baseline 2 DCE images

| Scan | UPN | File Path |
| --- | --- | --- |
| Baseline 1 | '01' | 'QIN-GBM-TR-01/05-07-1995-INVESTIGATORSCED-67244/16.000000-DCE2eco-41134' |
| Baseline 2 | '01' | 'QIN-GBM-TR-01/05-09-1995-INVESTIGATORSCED-49866/13.000000-DCE2eco-60143' |
| Baseline 1 | '03' | 'QIN-GBM-TR-03/06-10-1995-INVESTIGATORSCED-85079/16.000000-DCE2eco-89867' |
| Baseline 2 | '03' | 'QIN-GBM-TR-03/06-14-1995-INVESTIGATORSCED-63500/16.000000-DCE2eco-12831' |
| Baseline 1 | '05' | 'QIN-GBM-TR-05/06-24-1995-INVESTIGATORSCED-77209/18.000000-DCE2eco-36599' |
| Baseline 2 | '05' | 'QIN-GBM-TR-05/06-28-1995-INVESTIGATORSCED-01768/12.000000-DCE2eco-65522' |
| Baseline 1 | '06' | 'QIN-GBM-TR-06/06-20-1995-INVESTIGATORSCED-27178/17.000000-DCE2eco-41601' |
| Baseline 2 | '06' | 'QIN-GBM-TR-06/06-23-1995-INVESTIGATORSCED-76002/16.000000-DCE2eco-44204' |
| Baseline 1 | '08' | 'QIN-GBM-TR-08/07-05-1995-INVESTIGATORSCED-04644/17.000000-DCE2eco-28575' |
| Baseline 2 | '08' | 'QIN-GBM-TR-08/07-07-1995-INVESTIGATORSCED-09294/13.000000-DCE2eco-06434' |
| Baseline 1 | '09' | 'QIN-GBM-TR-09/07-12-1995-INVESTIGATORSCED-78304/17.000000-DCE2eco-39570' |
| Baseline 2 | '09' | 'QIN-GBM-TR-09/07-14-1995-INVESTIGATORSCED-48820/16.000000-DCE2eco-12784' |
| Baseline 1 | '10' | 'QIN-GBM-TR-10/08-06-1995-INVESTIGATORSCED-29384/14.000000-DCE2eco-06412' |
| Baseline 2 | '10' | 'QIN-GBM-TR-10/08-10-1995-INVESTIGATORSCED-74883/16.000000-DCE2eco-45526' |
| Baseline 1 | '11' | 'QIN-GBM-TR-11/10-01-1995-INVESTIGATORSCED-08116/17.000000-DCE2eco-55561' |
| Baseline 2 | '11' | 'QIN-GBM-TR-11/10-04-1995-INVESTIGATORSCED-07328/17.000000-DCE2eco-64920' |
| Baseline 1 | '12' | 'QIN-GBM-TR-12/10-11-1995-INVESTIGATORSCED-95009/15.000000-DCE2eco-93493' |
| Baseline 2 | '12' | 'QIN-GBM-TR-12/10-13-1995-INVESTIGATORSCED-78120/15.000000-DCE2eco-41460' |
| Baseline 1 | '13' | 'QIN-GBM-TR-13/10-21-1995-INVESTIGATORSCED-97643/17.000000-DCE2eco-94049' |
| Baseline 2 | '13' | 'QIN-GBM-TR-13/10-25-1995-INVESTIGATORSCED-52594/15.000000-DCE2eco-24465' |
| Baseline 1 | '14' | 'QIN-GBM-TR-14/11-07-1995-INVESTIGATORSCED-15850/14.000000-DCE2eco-26788' |
| Baseline 2 | '14' | 'QIN-GBM-TR-14/11-09-1995-INVESTIGATORSCED-01506/20.000000-DCE2eco-13784' |
| Baseline 1 | '17' | 'QIN-GBM-TR-17/12-08-1995-INVESTIGATORSCED-71894/15.000000-DCE2eco-73869' |
| Baseline 2 | '17' | 'QIN-GBM-TR-17/12-12-1995-INVESTIGATORSCED-44447/15.000000-DCE2eco-45169' |
| Baseline 1 | '18' | 'QIN-GBM-TR-18/12-29-1995-INVESTIGATORSCED-26938/24.000000-DCE2eco-44549' |
| Baseline 2 | '18' | 'QIN-GBM-TR-18/01-02-1996-INVESTIGATORSCED-99779/21.000000-DCE2eco-07031' |
| Baseline 1 | '20' | 'QIN-GBM-TR-20/01-28-1996-INVESTIGATORSCED-59688/21.000000-DCE2eco-81849' |
| Baseline 2 | '20' | 'QIN-GBM-TR-20/01-31-1996-INVESTIGATORSCED-85189/21.000000-DCE2eco-77213' |
| Baseline 1 | '21' | 'QIN-GBM-TR-21/02-02-1996-INVESTIGATORSCED-46481/21.000000-DCE2eco-75842' |
| Baseline 2 | '21' | 'QIN-GBM-TR-21/02-07-1996-INVESTIGATORSCED-14320/20.000000-DCE2eco-82624' |
| Baseline 1 | '23' | 'QIN-GBM-TR-23/03-08-1996-INVESTIGATORSCED-30393/25.000000-DCE2eco-61134' |
| Baseline 2 | '23' | 'QIN-GBM-TR-23/03-13-1996-INVESTIGATORSCED-91586/35.000000-DCE2eco-56604' |
| Baseline 1 | '25' | 'QIN-GBM-TR-25/03-23-1996-INVESTIGATORSCED-70993/23.000000-DCE2eco-73512' |
| Baseline 2 | '25' | 'QIN-GBM-TR-25/03-27-1996-INVESTIGATORSCED-25492/25.000000-DCE2eco-13945' |
| Baseline 1 | '40' | 'QIN-GBM-TR-40/03-26-1997-INVESTIGATORSCED-23153/27.000000-DCE2eco-34579' |
| Baseline 2 | '40' | 'QIN-GBM-TR-40/03-27-1997-INVESTIGATORSCED-55454/28.000000-DCE2eco-78689' |
| Baseline 1 | '41' | 'QIN-GBM-TR-41/04-26-1997-INVESTIGATORSCED-07577/27.000000-DCE2eco-34514' |
| Baseline 2 | '41' | 'QIN-GBM-TR-41/04-30-1997-INVESTIGATORSCED-70943/28.000000-DCE2eco-11162' |
| Baseline 1 | '42' | 'QIN-GBM-TR-42/05-11-1997-INVESTIGATORSCED-44896/12.000000-DCE2eco-78883' |
| Baseline 2 | '42' | 'QIN-GBM-TR-42/05-14-1997-INVESTIGATORSCED-06862/27.000000-DCE2eco-35815' |
| Baseline 1 | '43' | 'QIN-GBM-TR-43/03-10-1995-INVESTIGATORSNatHx-91662/16.000000-DCE2eco-97707' |
| Baseline 2 | '43' | 'QIN-GBM-TR-43/03-15-1995-INVESTIGATORSNHX-13022/16.000000-DCE2eco-53646' |
| Baseline 1 | '45' | 'QIN-GBM-TR-45/04-16-1995-INVESTIGATORSNHX-31020/16.000000-DCE2eco-37814' |
| Baseline 2 | '45' | 'QIN-GBM-TR-45/04-19-1995-INVESTIGATORSNHX-25857/18.000000-DCE2eco-12245' |
| Baseline 1 | '46' | 'QIN-GBM-TR-46/10-15-1995-INVESTIGATORSNHX-73132/15.000000-DCE2eco-35154' |
| Baseline 2 | '46' | 'QIN-GBM-TR-46/10-18-1995-INVESTIGATORSNHX-39848/11.000000-DCE2eco-31868' |
| Baseline 1 | '47' | 'QIN-GBM-TR-47/11-25-1995-INVESTIGATORSNHX-84491/15.000000-DCE2eco-80464' |
| Baseline 2 | '47' | 'QIN-GBM-TR-47/11-28-1995-INVESTIGATORSNHX-00632/15.000000-DCE2eco-37066' |
| Baseline 1 | '49' | 'QIN-GBM-TR-49/01-27-1996-INVESTIGATORSNHX-12900/15.000000-DCE2eco-93548' |
| Baseline 2 | '49' | 'QIN-GBM-TR-49/01-31-1996-INVESTIGATORSNHX-96493/16.000000-DCE2eco-80074' |
| Baseline 1 | '52' | 'QIN-GBM-TR-52/06-08-1997-INVESTIGATORSFMISO-60660/32.000000-DCE2eco-22187' |
| Baseline 2 | '52' | 'QIN-GBM-TR-52/06-12-1997-INVESTIGATORSNHX-08004/14.000000-DCE2eco-77872' |
| Baseline 1 | '55' | 'QIN-GBM-TR-55/09-11-1997-INVESTIGATORSNHX-66337/17.000000-DCE2eco-99141' |
| Baseline 2 | '55' | 'QIN-GBM-TR-55/09-14-1997-INVESTIGATORSNHX-98220/17.000000-DCE2eco-24796' |
| Baseline 1 | '56' | 'QIN-GBM-TR-56/09-19-1997-INVESTIGATORSNHX-22697/29.000000-DCE2eco-13337' |
| Baseline 2 | '56' | 'QIN-GBM-TR-56/09-20-1997-INVESTIGATORSNHX-79612/29.000000-DCE2eco-18155' |
| Baseline 1 | '57' | 'QIN-GBM-TR-57/11-06-1997-INVESTIGATORSNHX-43782/25.000000-DCE2eco-20805' |
| Baseline 2 | '57' | 'QIN-GBM-TR-57/11-08-1997-INVESTIGATORSNHX-41607/15.000000-DCE2eco-46677' |

**Table S11**: Summary of Test and Retest raw parameter values (mean ± standard deviation), for all observers, models, and VIF methods.

| **VIF Method** | **Model** | ***K^trans^***  **(min^-1^)** | | ***v_e_*** | | ***v_p_*** | | $\boldsymbol{\lambda}^{\boldsymbol{tr}}$  **(min^-1^)** | | **Tumor Volume**  **(n-voxels)** | |
| --- | --- | --- | --- | --- | --- | --- | --- | --- | --- | --- | --- |
|  |  | Test | Retest | Test | Retest | Test | Retest | Test | Retest | Test | Retest |
| **Manual**  **VIF** | Obs. 1  LTKM | 0.15  ±  0.075 | 0.15  ±  0.066 | 0.058  ±  0.029 | 0.053  ±  0.025 | 0.018  ±  0.0082 | 0.020  ±  0.010 | 0.0090  ±  0.0054 | 0.0087  ±  0.0040 | 2300  ±  1500 | 2300  ±  1500 |
|  | Obs. 1  eTM | 0.12  ±  0.056 | 0.12  ±  0.067 | 0.23  ±  0.077 | 0.25  ±  0.12 | 0.032  ±  0.017 | 0.034  ±  0.016 | - | - | 2100  ±  1300 | 2100  ±  1400 |
|  | Obs. 2  eTM | 0.076  ±  0.036 | 0.070  ±  0.029 | 0.074  ±  0.034 | 0.066  ±  0.031 | 0.0082  ±  0.0069 | 0.0051  ±  0.0033 | - | - | 3100  ±  1800 | 3000  ±  1900 |
| **Automatic**  **VIF** | Obs. 1  LTKM | 0.21  ±  0.088 | 0.22  ±  0.13 | 0.068  ±  0.031 | 0.072  ±  0.039 | 0.014  ±  0.0055 | 0.015  ±  0.0059 | 0.016  ±  0.0080 | 0.016  ±  0.0093 | 2500  ±  1500 | 2300  ±  1500 |
|  | Obs. 1  eTM | 0.11  ±  0.047 | 0.11  ±  0.053 | 0.10  ±  0.035 | 0.11  ±  0.042 | 0.014  ±  0.0047 | 0.015  ±  0.0052 | - | - | 2400  ±  1400 | 2300  ±  1400 |
|  | Obs. 2  eTM | 0.096  ±  0.044 | 0.072  ±  0.033 | 0.12  ±  0.040 | 0.12  ±  0.073 | 0.010  ±  0.0078 | 0.0082  ±  0.0041 | - | - | 3100  ±  1800 | 3000  ±  1900 |
